# Tea in Health and Disease

**DOI:** 10.3390/nu11040929

**Published:** 2019-04-25

**Authors:** Q. Ping Dou

**Affiliations:** Barbara Ann Karmanos Cancer Institute and Departments of Oncology, Pharmacology and Pathology, School of Medicine, Wayne State University, Detroit, MI 48201-2013, USA; doup@karmanos.org; Tel.: +313-576-8301

Tea, including green tea made from the leaves of the *Camellia senenisis* plant, is the second most consumed beverage worldwide after water, and is consumed by more than two-thirds of the world population [1,2,3]. Accumulating evidence from cellular, animal, clinical and epidemiological studies have linked tea consumption to various health benefits, such as chemoprevention of cancers, chronic inflammation, heart and liver diseases, diabetes, neurodegenerative diseases, ultraviolet B (UVB)-induced skin aging, bone fracture, etc., along with some other beneficial activities, e.g., chemo-sensitizing, antioxidizing stress-reducing, etc. [1,2,3]. Although some of these health benefits have not been consistently achieved by intervention trials, positive results from some clinical trials have provided direct evidence supporting the protective effect of tea against, at least, human cancer [1,2,3,4,5]. In addition, multiple mechanisms of action have been proposed to explain how tea exerts its disease-preventive effects. 

This special issue of Nutrients, “*Tea in Health and Disease*”, has collected nine (9) research articles and four (4) comprehensive review articles. All of these publications are timely, novel, and written by authors who are experts in the field of tea research. 

Jang, Hwang and Choi found in their research article, that rosmarinic acid, a compound isolated from rosemary tea, modulates expression of histone deacetylase 2 and inhibits growth of prostate cancer cells via induction of the cell cycle arrest and apoptosis [6].

Heyza et al., reported, in their original study, that green tea polyphenol (–)-epigallocatechin-3-gallate (EGCG) acts as a potent inhibitor of the 5′-3′ structure-specific endonuclease ERCC1/XPF (Excision Repair Cross-Complementation Group 1/Xeroderma Pigmentosum Group F) in human cancer cells, serving as an ideal candidate for further pharmacological development with the goal of enhancing cisplatin response in human tumors [7].

Farabegoli et al., discovered that the combinational treatment of EGCG and a rexinoid, 6-OH-11-O-hydroxyphenanthrene [IIF] inhibits neuroblastoma cell growth and neurosphere formation in vitro [8]; the authors concluded that the association of EGCG to IIF might be able to overcome the incomplete success of retinoid treatments in neuroblastoma patient without toxic effects.

Zhao et al., reported that Fuzhuan brick-tea protects against UVB irradiation-induced photo-aging via MAPKs/Nrf2-mediated down-regulation of MMP-1, and suggested that this tea could be used as not only a functional food but also a good candidate in the development of cosmetic products and medicines for the remedy of UVB-induced skin photo-aging [9].

Annunziata et al., evaluated colon bioaccessibility and antioxidant activity of tea polyphenolic extract by using an in vitro simulated gastrointestinal digestion assay [10]. They found that after gastrointestinal digestion, the bioaccessibility and the antioxidant activity in the colon stage were significantly increased compared to the duodenal stage for both tea polyphenols and total phenol content. These results could be attributable in vivo to the activity of gut microbiota, which metabolize tea compounds and generate metabolites with a greater antioxidant activity [10].

Pan et al., report that polyphenols in Liubao tea prevent carbon tetrachloride-induced hepatic damage in mice through their antioxidant function [11]. Molecularly, Liubao tea modulates various enzymatic activities and reduces serum levels of several cytokines in mice with liver injury. 

Shen et al., examined the association between tea consumption and risk of hospitalized fracture in 453,625 Chinese adults. Their study concluded that habitual tea consumption was associated with moderately decreased risk of any fracture hospitalizations, and the participants with decades of tea consumption and those who preferred green tea were also associated with lower risk of hip fracture [12].

Unno et al., determined the stress-reducing function of matcha green tea (that contain high levels of theanine, a major amino acid) in both animal experiments and clinical trials [13]. They found that high contents of theanine and arginine in matcha exhibited a high stress-reducing effect in mice, and that anxiety, a reaction to stress, was significantly lower in the matcha tea-consuming participants than in the placebo group.

Rode et al., determined, in a cross-sectional observational study among a population of 273 hypercalciuric stone-formers, whether daily green tea drinkers experienced increased stone risk factors (especially for oxalate) compared to non-drinkers, and found no evidence for increased stone risk factors or oxalate-dependent stones in daily green tea drinkers [14].

Furthermore, Khan and Mukhtar extensively reviewed the health-promoting effects of tea polyphenols [15], by summarizing recent studies on the role of tea polyphenols in the prevention of cancer, diabetes, cardiovascular and neurological diseases. Negri et al., presented another comprehensive updated summary on molecular targets of green tea polyphenol EGCG with a special focus on the involved signal transduction pathways in human cancer [16].

In another review article, Gan et al., summarized the distribution, composition, and health benefits of several caffeinated beverages from the genus *Ilex*, including the large-leaved Kudingcha (*Ilex latifolia* Thunb and *Ilex kudingcha* C.J. Tseng), Yerba Mate (*Ilex paraguariensis* A. St.-Hil), Yaupon Holly (*Ilex vomitoria*), and Guayusa (*Ilex guayusa* Loes), and suggested their potential applications in the pharmaceutical or nutraceutical industries [17].

Tea consumption is also considered a natural complementary therapy for neurodegenerative diseases such as Alzheimer’s disease that affects an increasing patient population among the elderly. Polito et al., reviewed epidemiological studies on the association between tea consumption and the reduced risk of Alzheimer’s disease, along with the anti-amyloid effects and the role of tea in preventing this neurodegenerative disease [18].

While beneficial effects by tea consumption have been documented in various human disease models as mentioned above, there are major challenges in developing some tea components (such as green tea polyphenols) as therapeutic agents, including how to improve their bioavailabilities, stability, efficacies, and specificity [5]. Further well-designed preclinical and clinical studies are warranted in the future. 

I would like to thank all the authors for their exceptional contributions. 
